# The Role of Angiogenesis in Breast Cancer and Obesity: Unravelling the Connection

**DOI:** 10.1155/ijbc/2364200

**Published:** 2026-06-20

**Authors:** Yolo O. K. Sikuza, Charlise Basson, Nanette Oberholzer, Melvin A. Ambele

**Affiliations:** ^1^ Department of Immunology, Institute for Cellular and Molecular Medicine, and SAMRC Extramural Unit for Stem Cell Research and Therapy, Faculty of Health Sciences, University of Pretoria, Pretoria, South Africa, up.ac.za; ^2^ Department of Oral and Maxillofacial Pathology, Faculty of Health Sciences, School of Dentistry, University of Pretoria, Pretoria, South Africa, up.ac.za; ^3^ Department of Anatomy, Faculty of Health Sciences, University of Pretoria, Pretoria, South Africa, up.ac.za

**Keywords:** angiogenesis, breast cancer, endothelial cells, hypoxia, obesity, VEGF

## Abstract

Breast cancer remains one of the leading causes of cancer‐related mortality amongst women worldwide, with rising incidence rates paralleling the global obesity epidemic. Obesity has been increasingly recognised as a risk factor for breast cancer, yet the molecular mechanisms underlying the association remain poorly understood. This review explores the role of angiogenesis as the central mechanism linking obesity to breast cancer progression. Angiogenesis is essential for both adipose tissue expansion and tumour growth. It is dysregulated in obesity and breast cancer, resulting in the formation of abnormal vasculature that perpetuates hypoxia and malignancy. Obesity contributes to this process through hypertrophic adipose tissue, altered adipokine profiles and elevated expression of proangiogenic factors, such as VEGF. These changes create a tumour microenvironment conducive to cancer progression, treatment resistance and poor clinical outcomes. Emerging evidence also implicates endothelial cells, pericytes and lipid metabolism in this interaction, suggesting novel therapeutic targets.

## 1. Introduction

Angiogenesis is the formation of new blood vessels from already existing ones to supply oxygen and nutrients to different tissues [1]. It is highly controlled by the equilibrium between proangiogenic and antiangiogenic factors [2]. Angiogenesis plays a key role in the expansion of both adipocytes [3] and tumour cells, with the effect on the latter being increased proliferation, progression and metastasis [4]. Tumour cells are surrounded by a dense capillary network in order to provide necessary nutrients and oxygen to the cells [5]. In breast cancer, angiogenesis is dysregulated, causing an increase in the release of proangiogenic factors, leading to an increase in angiogenesis and excessive tissue expansion [6]. This increase in proangiogenic factors is associated with a decrease in breast cancer patient survival [7].

Breast cancer is one of the most common cancers affecting women globally. It is characterised by the uncontrolled growth of abnormal breast tissue, which can form into tumours that invade surrounding tissues [8]. Although breast cancer can occur in men, it is significantly more common in women [9]. Breast cancer accounts for approximately one in four cancer diagnoses and one in six cancer deaths among women, making it the leading cause of cancer‐related mortality in women across most countries [10]. In contrast, breast cancer in men is rare, accounting for 1% of all breast cancer cases [11]. During the 1980s and 1990s, the incidence of breast cancer rose largely due to increased screening [12]. Since 2007, the incidence has continued to rise [12], and if current trends persist, breast cancer cases are projected to exceed 3 million, with an estimated 1 million deaths by 2040 [13]. The most recent statistics in 2026 reported that approximately 2.3 million women were diagnosed with breast cancer worldwide, resulting in approximately 764,000 deaths in 2023 [14]. In the United States, projections for 2026 indicate that around 321,910 women will be diagnosed with invasive breast cancer. In men, the incidence remains consistently low. Amongst women, about 16% of breast cancer diagnoses occur in those younger than 50 years of age, and approximately 42,140 women will succumb to this disease in 2026 [15].

Breast cancer is a heterogeneous disease with multiple subtypes, each with distinct biological and clinical characteristics. The most common subtypes include Luminal A, Luminal B, human epidermal growth factor Receptor 2 (HER2)–positive and triple‐negative breast cancer (TNBC). These classifications are based on the expression of hormone receptors for oestrogen receptor (ER), progesterone receptor (PR) and HER2 [16, 17]. The Luminal A subtype is characterised by ER and/or PR positivity, HER2 negativity and low Ki67 expression. This profile reflects slower cell proliferation and growth, making Luminal A generally low grade with a more favourable prognosis and higher survival rates [18]. In contrast, the Luminal B subtype is also ER‐positive but may be PR‐negative; it typically exhibits high Ki67 expression [19]. This increases the cell proliferative activity and results in a worse prognosis than Luminal A, with a higher recurrence rate [20]. The HER2‐positive subtype is defined by a high expression of HER2 whilst being negative for both ER and PR. These tumours are more aggressive and fast‐growing, compared to Luminal A and B [21, 22]. The TNBC is characterised by a lack of ER, PR and HER2 expression and accounts for approximately 10%–15% of breast cancers worldwide. It is highly aggressive and has a rapid proliferation rate, alterations in DNA repair genes and a greater tendency to present at advanced stages [21]. Excess weight is a well‐established risk for postmenopausal breast cancer, particularly hormone receptor–positive (ER+/PR+) subtypes; however, its influence across all breast cancer subtypes is nuanced, varying with menopausal status, and not uniformly affecting each type [23].

Given the increase in incidence in recent years, the influence of metabolic conditions such as obesity has been under scientific scrutiny. The rising rates of obesity have been accompanied by an observable increase in breast cancer cases [24]. Breast cancer has a complex aetiology; understanding the diverse risk factors, such as obesity, is essential to understanding breast cancer development, progression and the management thereof. Much like tumour tissue, adipose tissue is highly vascular, and angiogenesis is necessary for this vascularisation [25]. The increase in angiogenic activity observed in both obesity and breast cancer indicates a potential mechanistic link between these two conditions.

Obesity arises from a sustained caloric surplus, resulting in weight gain [26]. On a cellular level, obesity is characterised by adipocyte expansion, which can either manifest as hypertrophy or hyperplasia [27]. In adipocyte hypertrophy, adipocytes are enlarged to accommodate the caloric surplus stored as lipid, whilst adipocyte hyperplasia is characterised by an increase in the number of adipocytes [28]. The clinical classification of obesity relies on body mass index (BMI) measurements, with a BMI of 30 kg/m^2^ and above classified as obesity and a BMI of 25–29.9 kg/m^2^ as overweight [29]. Beyond the accumulation of excess body fat, obesity contributes to a range of adverse health outcomes [30], including cancer [31]. The prevalence of obesity‐related complications has increased drastically over the past decades [32]. In the context of breast cancer, obesity is associated with an increased risk of breast cancer development and poorer clinical outcomes [33]. Although the association between breast cancer and obesity has been established, the underlying molecular mechanisms remain unknown. Studies report conflicting effects of obesity on breast cancer, with both adverse and protective associations observed across different studies [34]. One study reported that the 5‐year survival rate for breast cancer was 55.6% amongst obese or overweight women, compared to 79.9% in women of normal weight. The study further highlighted a correlation between obesity and a greater likelihood of larger tumour sizes and the presence of invasive lymph nodes [35]. Contrasting evidence revealed that obesity has protective benefits in premenopausal women [36, 37]. A study found a 4.2‐fold increase in breast cancer risk in underweight women versus obese women [38]. Nevertheless, obesity may alter fundamental hallmarks of cancer [39], including angiogenesis.

Understanding how obesity promotes breast cancer progression through angiogenesis may lead to the identification of novel biomarkers and improved therapeutic strategies that target angiogenesis in cancer treatment. Since breast cancer is one of the most common causes of cancer‐related deaths amongst women, understanding how obesity impacts it, specifically at a molecular level through the lens of angiogenesis, can have important public health implications. This review, therefore, is aimed at examining current evidence on the molecular link between obesity and breast cancer with a focus on angiogenesis.

## 2. Angiogenesis

**Figure 1:**
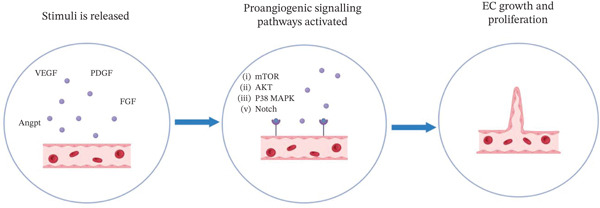
A summary of angiogenesis and pathways stimulated on endothelial growth and proliferation. This image was created using BioRender (https://http://www.biorender.com/). AKT, protein Kinase B; Angpt, angiopoietin; EC, endothelial cell; FGF, fibroblast growth factor; MAPK, mitogen‐activated protein kinase; mTOR, mechanistic target of rapamycin; PDGF, platelet‐derived growth factor; VEGF, vascular endothelial growth factor.

Angiogenesis is a complex process that involves the activation of endothelial cells (ECs), degradation of the extracellular matrix, proliferation and migration of ECs and tubular formation [40]. The blood vessels usually do not proliferate unless the ECs, which line the blood vessels, are stimulated to multiply [7]. Under physiological conditions, angiogenesis can be stimulated by hypoxia, amongst other metabolic signals, which include glycolysis [41], increased lactate [42] or high levels of glucose [43]. When stimulated, key proangiogenic factors are released, including vascular endothelial growth factor (VEGF), platelet‐derived growth factor (PDGF), fibroblast growth factor (FGF) and angiopoietins (Angpt) [44]. This stimulates signalling pathways that promote cell survival, proliferation and migration of ECs [45]. These pathways include mechanistic target of rapamycin (mTOR) [45], which is responsible for the regulation of growth, metabolism and protein synthesis [46]. The protein Kinase B (AKT) pathway [45] is known to enhance the survival, proliferation and migration of ECs [47]. Proangiogenic factors also stimulate the p38 mitogen‐activated protein kinase (p38 MAPK) and the Notch signalling pathway (Figure 1) [45]. In a pathological setting, the chronic inflammation and hypoxia lead to a proangiogenic state contributing to diseases such as cancers and obesity [48].

### 2.1. Angiogenesis in Breast Cancer

During the beginning stages of tumour development, angiogenesis does not occur until the activation of the angiogenic switch, which defines a critical turning point in tumour development where previously dormant, nonvascularised tumour begins to actively recruit new blood vessels to support tumour growth [49]. Once the tumour grows beyond 1–2 mm in diameter, the blood supply becomes insufficient, triggering the angiogenic switch [2, 50]. Vascular homeostasis is a balance between proangiogenic and antiangiogenic factors, which are secreted by the tumour, immune cells and stromal components [2, 50], such as adipocytes [51].

The rapid proliferation of a tumour creates a hypoxic, glucose‐deprived environment that initiates the release of proangiogenic factors VEGF, FGF and PDGF from tumour cells [52]. When the proangiogenic factors outweigh the antiangiogenic factors, it creates a dominant stimulus [50]. The release of these proangiogenic growth factors stimulates quiescent ECs to sprout and migrate towards the tumour [52]. Vascularisation of tumours also allows for cancer cells to have a route for metastasising to other organs [53]. However, the newly formed blood vessels in tumours are often immature and poorly structured, leading to further hypoxia and acidic conditions within the tumour microenvironment (TME) [54]. These conditions promote further tumour growth and metastasis, creating a self‐perpetuating cycle [54]. Other than cancers, metabolic conditions such as obesity have been seen to further amplify angiogenic signalling [55], which can influence cancer progression.

### 2.2. Angiogenesis in Obesity

Adipose tissue angiogenesis plays a crucial role in maintaining adipose function and promoting adipogenesis. It is essential for nutrients and oxygen supply, as well as aiding the endocrine function of adipose tissue [56]. In obesity, adipose tissue becomes hypertrophic, leading to a decrease in oxygen supply to adipocytes due to insufficient blood vessels in the expanded adipose tissue [3]. The vascular system influences the adipose tissue environment by managing acidosis and hypoxia, which impact the behaviour and formation of adipocytes [3]. Angiogenesis is often triggered by the expansion of adipocytes or changes in the metabolic signals, such as fatty acid oxidation and glycolysis [57]. As adipocytes expand and accumulate lipids in conditions of nutrient surplus, the oxygen levels drop, initiating hypoxia [58]. In a healthy state, adipose tissue blood vessels are lined with quiescent ECs, which can transition to a proliferative state in response to angiogenic signalling [57]. The adipose tissue also produces angiogenic factors, such as VEGF and Angpt2, and other adipokines for blood vessel growth and structure [3]. Using in vivo imaging, a study found that blood flow in the epididymal adipose tissue of lean mice remained constant, even at the capillary level; meanwhile, blood flow in obese mice was irregular and intermittent [59]. These findings suggest impaired perfusion in hypertrophic adipose tissue, potentially leading to localised hypoxia [59].

Moreover, certain adipokines secreted by adipose tissue can function as proangiogenic factors, contributing to the regulation of vascular growth within the tissue [60]. In lean individuals, adipocytes release relatively low levels of leptin and elevated levels of adiponectin, the latter functioning as an antimitogenic factor [34, 61]. In obese individuals, there is a high number of immature adipocytes known as preadipocytes. These cells release high levels of leptin, a proangiogenic molecule, leading to an increase in angiogenesis [34, 61]. These alterations seen in obesity may influence the TME and tumour development.

### 2.3. Angiogenesis in Breast Cancer and Obesity

In obesity, the increase in leptin and cytokines upregulates the proangiogenic factor VEGF to stimulate breast tumour angiogenesis [61]. The production of leptin from the preadipocytes further stimulates angiogenesis [62]. An in vivo study using C57BL/6J mice revealed that postmenopausal mice fed a high‐fat diet (HFD) showed a higher density of microvessels, which are small blood vessels which play a vital role in maintaining vascular function [63], in their breast tumours compared to the control group [64]. A Phase II clinical trial evaluating antiangiogenesis therapy demonstrated that patients with a BMI greater than 25 kg/m^2^ had tumours that were on average 33% larger compared to those with a BMI below 25 [65]. Increased angiogenesis was seen in both obesity and breast cancer, often leading to poorly organised blood vessels that create hypoxic conditions within the tumour, thereby playing a major role in tumour progression [66].

## 3. Hypoxia

Hypoxia, characterised by low oxygen levels, is a hallmark feature of the TME [6]. It is important in the induction of angiogenesis in breast cancer [6] and adipogenesis [55]. Hypoxia stimulates an increase in proangiogenic factors once the tumour or adipocyte outgrows the current blood supply, causing a decrease in oxygen, leading to hypoxia [67, 68]. In response, the cells release hypoxia‐inducible factors (HIFs), which are the main transcription regulators for proangiogenic factors [69], including VEGF [7]. Other markers of hypoxia include Carbonic Anhydrase IX (CAIX) [70].

HIFs represent a family of transcription factors consisting of a heterodimer with both an oxygen‐related *α*‐subunit and a *β*‐subunit, which is continuously expressed [71]. During normoxic conditions, the HIF‐*α* subunit is degraded through hydroxylation. However, in hypoxic conditions, the hydroxylation is inhibited [72], which stabilises the HIF‐*α* unit to move into the nucleus, forming a dimer with the HIF‐*β* subunit and coactivators such as CBP/p300 [72]. The heterodimers then activate the hypoxia‐responsive elements, resulting in their transcription [73]. Three different forms of *α*‐subunits have been reported, namely, HIF‐1*α*, HIF‐2*α* and HIF‐3*α* [74]. Hypoxia‐inducible factor‐1 stimulates angiogenesis through proangiogenic factors, such as VEGF and PDGF, which are targets for HIF‐1 [75]. A combination of patient observational and in vitro studies has found the expression of HIF‐1*α* and HIF‐2*α* to be elevated in breast cancer, which has been linked to a lower survival rate [76–79].

### 3.1. Hypoxia in Breast Cancer

Hypoxia is prominent in aggressive breast cancer subtypes such as TNBC and basal‐like breast cancers, contributing to their poor prognosis and high metastatic potential [80, 81]. Recent studies have consistently shown a strong link between hypoxia and key aspects of cancer development, including tumour initiation, metastasis, resistance to treatment and increased patient mortality [82–84]. Hypoxic zones are found in approximately 25%–40% of invasive breast cancers [85]. These oxygen‐deficient areas within tumours and their surrounding microenvironment typically arise from irregular blood vessel architecture, excessive angiogenesis that causes localised blockages or pressure and impaired microcirculatory function [86].

Different subtypes of breast cancer have varying degrees of hypoxia. The Luminal A subtypes exhibit lower levels of hypoxia due to better vascularisation, whilst Luminal B is prone to the development of hypoxic regions. In human epidermal growth receptor‐3 cancers, HIF‐1*α* and HIF‐2*α* are frequently activated due to fast‐growing tumours [87]. TNBCs, which lack ER, exhibit the highest degree of hypoxia, characterised by elevated expression of HIF‐1*α*, HIF‐2*α* and CAIX. The presence of hypoxia in TNBC has been associated with poor patient prognosis and aggressive tumours [87]. A study found that TNBC patients had significantly higher HIF‐1*α* expression, which was coupled with increased VEGF‐A expression when compared to the other cancer subtypes [88]. In TNBC, increased hypoxia, coupled with increased VEGF‐A expression, suggests that angiogenesis may play a central role in driving tumour aggressiveness [80, 88]. Consequently, further exacerbation of hypoxic conditions, driven by alterations within the TME, could intensify the pathological effects associated with angiogenic activity.

### 3.2. Hypoxia in Obesity

In obesity, adipocytes can expand beyond 200 *μ*m in diameter, surpassing the natural oxygen diffusion limit within tissues and increasing the need for tissue vascularisation [3, 68, 89]. This excessive enlargement leads to insufficient oxygen delivery, resulting in local hypoxia [68], which triggers an inflammatory response activated by tumour necrosis‐*α*, interleukin‐6 and monocyte chemotactic protein‐1 [90]. Studies using ob/ob mouse models with diet‐induced obesity have demonstrated that mice with obesity exhibit significantly reduced oxygen partial pressure (pO_2_), with values ranging from 8 to 20 mmHg, which is considerably lower than the 27–40 mmHg observed in lean mice [91–93]. Clinical studies further support this finding, showing a decline in adipose tissue pO_2_ to approximately 47 mmHg in individuals with obesity, compared to 55 mmHg in lean individuals [68]. In another study, adipocytes isolated from mice on an HFD and lean mice showed that the HFD mice had an increased rate of oxygen consumption [94], indicating possible hypoxia. As the adipose tissue continues to expand, the formation of new blood vessels through angiogenesis cannot keep up with the tissue expansion. This causes a reduction in blood flow and circulation of nutrients and waste removal in these tissues [3]. Similar to tumour development in cancer, the expansion of adipose tissue under hypoxic conditions follows a comparable biological pattern. The shared hypoxic response in breast cancer and obesity may create an environment that promotes cancer development and progression. Collectively, these observations show how hypoxia‐driven angiogenesis in expanding adipose tissue mirrors tumour biology, revealing a convergent pathway where impaired oxygen may potentially facilitate cancer progression.

### 3.3. Hypoxia in Breast Cancer and Obesity

**Figure 2:**
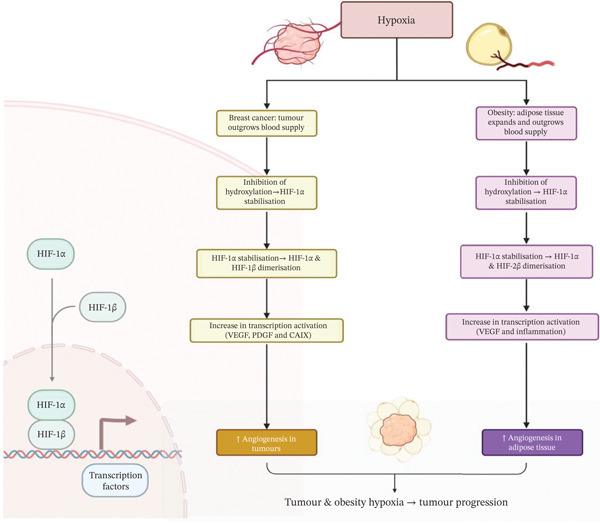
Illustration of hypoxia in breast cancer and obesity. This image was created using BioRender (https://www.biorender.com/). CAIX, Carbonic Anhydrase IX; HIF, hypoxia‐inducible factor; PDGF, platelet‐derived growth factor; VEGF, vascular endothelial growth factor.

Adipocytes and the TME both create hypoxic environments [95] due to the expansion [96] and proliferation of cells [97]. The overlap in hypoxic signalling suggests a potential synergistic interaction or even a cause‐and‐effect relationship [95], where adipocyte‐driven metabolic stress may intensify the hypoxic state of TME, further promoting progression. An in vitro study cocultured MCF‐7 breast cancer cells and human adipocytes showed a reduction of ER‐*α* in MCF‐7 cells, which was associated with a higher expression of the HIF‐1*α* gene in the hypoxic adipocytes, thus decreasing the therapeutic effects of hormone treatment [98]. However, studies have found that lower levels of ER‐*α* in breast cancer cells are linked to lower angiogenic activity, as ER‐*α* is a driver of angiogenesis [99]. These findings suggest that adipocyte‐induced hypoxia may not only alter hormone receptor signalling but also intensify hypoxia within tumour cells, further influencing tumour progression. In vitro coculture of adipocytes and other cancer cell types has been shown to increase CAIX expression [100], suggesting that adipocytes actively modulate the TME. This interaction may have significant implications in breast cancer, where adipocyte‐driven hypoxic signalling could contribute to tumour progression. Together, these findings highlight the dynamic role of adipocytes in influencing the TME (Figure 2).

## 4. Angiogenic Factors

Growth and metastasis of the tumour are highly dependent on the angiogenic process [7]. The release of proangiogenic factors and Angpt, stimulated by hypoxia, which is a physiological state, triggers angiogenesis [6]. Multiple pathways, including FGF, PDGF and transforming growth factor‐beta (TGF‐*β*), affect angiogenesis [6] by influencing EC behaviour and interacting with the tumour and the TME [4, 6]. However, the central angiogenic signalling pathway is the VEGF [44].

### 4.1. VEGF

**Figure 3:**
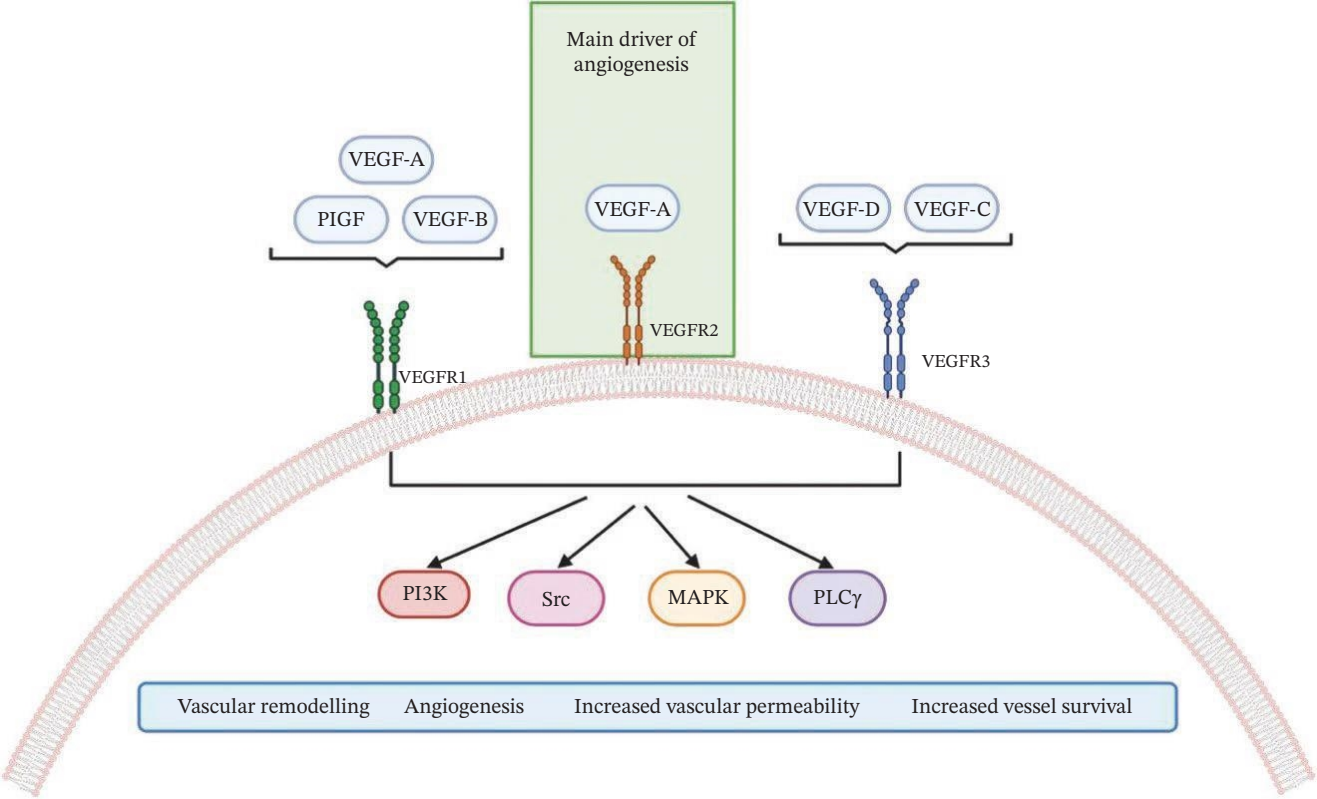
VEGF activation and stimulation of the downstream signalling pathways. This image was created using BioRender (https://www.biorender.com/). MAPK, mitogen‐activated protein kinase; PI3K, phosphatidylinositol 3‐kinase pathway; PLC*γ*, Phospholipase C gamma.

VEGF is a crucial regulator of angiogenesis and stimulates the proliferation of ECs [101]. These signalling proteins play a significant role in new blood vessel formation. The VEGF family includes several proteins: VEGF‐A, VEGF‐B, VEGF‐C, VEGF‐D and placental growth factor (PIGF) [102]. These proteins form homodimers or heterodimers and bind to the tyrosine kinase receptors on the cell surface [102, 103]. Once the VEGF binds to its respective receptors, they trigger downstream pathways including the phosphatidylinositol 3‐kinase (PI3K), cytoplasmic tyrosine kinase Src, mitogen‐activated protein kinase (MAPK) and Phospholipase C gamma (PLC*γ*) [102, 104]. The function of the proteins varies with VEGF‐A promoting angiogenesis, vascular permeability, vascular remodelling and vessel survival [102]. The protein mainly found in blood vessel ECs is VEGF‐A, and it binds to vascular endothelial growth factor Receptor (VEGFR) 1 and VEGFR‐2 [102]. The release of VEGF binds to receptors on ECs and stimulates proliferation and, therefore, blood vessel formation (Figure 3) [6]. VEGF‐A is found to be upregulated in many cancers, including breast cancer [105].

#### 4.1.1. VEGF in Breast Cancer

Elevated VEGF expression correlates with increased microvessel formation [106] and is related to advanced stages of disease and poor patient prognosis [107, 108]. Compared to normal or benign breast tissue, breast cancer is associated with significantly high levels of VEGF transcripts [105]. A study involving 30 females with benign breast lesions found that VEGF serum levels were elevated in those diagnosed with breast cancer before surgery, in contrast to their VEGF levels following the removal of benign lesions [109]. These findings suggest that VEGF expression in breast cancer is not limited to transcriptional changes but also manifests at the physiological level, indicating a systemic angiogenic response.

Within the TME, ECs and smooth muscle cells actively secrete VEGF [110, 111]. The tumours release VEGF‐A to stimulate the nearby ECs, driving the formation of new blood vessels [112]. In most cancers, VEGF‐A is upregulated, fuelling the growth of morphologically abnormal blood vessels. It disrupts the endothelial barrier by activating Src kinase, which disrupts the VE‐cadherin/*β*‐catenin complex, weakening the vessel walls, which enables cells to escape to surrounding tissues [113]. Taken together, these findings illustrate VEGF′s central role in promoting tumour angiogenesis and highlight its potential as a target for therapeutic intervention in breast cancer.

#### 4.1.2. VEGF in Obesity

In obesity, VEGF expression is elevated and influenced by multiple factors associated with tissue dysfunction. One such factor is leptin, a hormone whose levels are significantly increased in obesity. Elevated leptin has been linked to obesity‐related cancers, including breast cancer [114]. In this context, dysregulation of leptin signalling can promote tumour growth both directly and indirectly by enhancing angiogenesis and neovascularisation [115]. A study using 4T1 breast cancer cells demonstrated that leptin treatment enhanced VEGF production and activity. Specifically, leptin increased VEGF secretion in a dose‐dependent manner and activated the VEGF gene promoter. This resulted in a threefold increase in gene activity, and leptin further elevated VEGFR‐2 [116].

Another contributor is fatty acid synthase (FASN), a central enzyme in lipid metabolism. The expression of FASN is upregulated during adipogenesis, particularly in response to high‐calorie diets [117, 118]. In obesity, FASN activity is further increased to manage the excessive nutrients [118]. Notably, in breast cancer models such as MCF‐7‐MEK cells, both in vitro and in vivo studies have shown an increase in FASN expression, which was accompanied by elevated levels of VEGF and VEGFR‐2, which are key mediators of angiogenesis [119]. These findings suggest that the increase in adipogenesis observed in obesity may contribute to increased VEGF expression, potentially through FASN‐driven pathways that promote angiogenic signalling.

Supporting this relationship are two independent in vivo studies that have highlighted the role of VEGF in obesity‐related angiogenesis. One study reported a 100% increase in VEGF mRNA levels in obese mice, which correlated with elevated VEGF protein expression in both diet‐induced and genetically obese mice [120]. Another study demonstrated that overexpression of VEGF in adipose tissue leads to an increase in both the number and size of blood vessels, suggesting that VEGF expression in adipocytes may promote angiogenesis [121].

Interestingly, not all VEGF isoforms promote angiogenesis. An ex vivo study found that VEGF‐A_165_b, an antiangiogenic isoform, was significantly increased in the visceral fat of obese individuals [122]. This upregulation was associated with reduced capillary growth. Whilst hypoxia in adipose tissue typically induces proangiogenic factors such as VEGF‐A_165_b, dysregulated signalling in obesity may instead favour antiangiogenic factors [123], such as VEGF‐A_165_b. Alternatively, these results may indicate that the tissue had not yet exceeded its vascular capacity, suggesting the absence of hypoxia. Overall, the findings suggest that elevated VEGF‐A_165_b levels could contribute to hypoxia and exacerbate adipose tissue dysfunction. Altogether, these findings reveal that obesity‐associated metabolic changes influence VEGF expression through various signalling pathways, intricately linking adipose tissue dysfunction with both pro‐ and antiangiogenic mechanisms that may influence cancer progression and vascular remodelling.

## 5. ECs

Blood vessels are lined with a thin layer of ECs. These cells are the primary cells responsible for angiogenesis. Angiogenesis happens in two main forms: intussusception and sprouting angiogenesis. In sprouting angiogenesis, the new blood vessels form from existing blood vessels through tree‐like branching. Intussusceptive angiogenesis involves the splitting of existing blood vessels [124]. Once angiogenesis is triggered, VEGF travels to the VEGFR‐2 on EC, and this interaction promotes EC proliferation, migration and survival through the activation of the PI3K/AKT and ERK pathways [125].

The ECs loosen their cell–cell interactions. Pericytes detach, and the basement membrane is degraded. This allows for a new capillary to form. The EC with the strongest VEGF signal becomes the tip cell. This cell migrates towards the VEGF source, extending long projections known as filopodia. The tip cell selection is reinforced by Notch–Delta signalling. The tip cell expresses Delta‐like Ligand 4 (Dll4), which activates Notch receptors in the neighbouring cells, causing a suppression in their tip cell potential, making them stalk cells. The surrounding ECs become stalk cells, which proliferate, form the body of the vessel and are responsible for the lumen formation. As the blood vessels form, pericytes and smooth muscles reattach to provide stability to the new blood vessel [126].

### 5.1. ECs in Breast Cancer

Tumour angiogenesis is often characterised by irregular tortuous blood vessels, disorganised ECs and poorly structured pericytes, which all contribute to increased vascular permeability [48, 127, 128]. These abnormalities in tumour vascularisation contribute to tissue metastasis, hinder immune cell infiltration and affect the effectiveness of therapeutic delivery [50, 127, 129–131]. Under normal physiological conditions, ECs remain in quiescence until stimulated by metabolic or angiogenic signals that trigger their proliferation [57, 132]. However, excessive expression of proangiogenic factors may contribute to EC dysfunction. Impaired ECs are unable to support proper angiogenesis, resulting in reduced blood vessel formation and potentially increasing hypoxia [25, 126].

A study using different types of breast tissues from patients found the presence of Dll4, a key component of the Notch signalling pathway, which helps regulate angiogenesis [133]. In cancer, Dll4 becomes abnormally active, promoting the development of dysfunctional blood vessels [133]. In this study, Dll4 was found near invasive breast cancer but not in nonlactating breast tissue. In contrast, lactating breast tissue showed high levels of Dll4, similar to invasive breast cancer [134]. These findings suggest that Dll4 expression may be closely linked to EC remodelling and angiogenic activity.

A study developed two immortalised human EC lines, one from healthy breast tissue and one from breast tumour tissue derived from the same patient. Both cell lines retained endothelial identity, expressing markers such as Von Willebrand factor, cluster of Differentiation 31, cluster of Differentiation 105, VEGFR‐2 and platelet EC adhesion molecule expression, indicating tumour‐induced phenotypic changes. Functional comparisons revealed that healthy ECs had higher proliferation rates, whilst tumour‐derived ECs exhibited stronger VEGF‐A induction under hypoxia, despite lower baseline secretion, reflecting their abnormal angiogenic response. Moreover, both cell lines could form capillary‐like structures in vitro, but healthy ECs formed more complete vascular networks compared to tumour‐derived ECs, which produced leaky and poorly organised vessels [135].

Together, these findings highlight the critical role EC integrity and molecular signalling play in the quality of angiogenesis and how tumour‐derived ECs can differ from healthy ECs in both structure and function. Interestingly, similar EC alteration may also occur in obesity, where chronic inflammation and adipose expansion demand vascular remodelling [136].

### 5.2. ECs in Obesity

Adipose tissue expansion involves several cells, including the ECs in the vasculature [55]. Obesity has been associated with EC dysfunction and reduced vascular density, largely driven by the overexpression of proangiogenic and proinflammatory molecules [3]. As metabolic needs increase, the body releases insulin‐like growth factor‐1 (IGF‐1) to regulate growth and metabolism [137]. The capillaries around adipocytes express IGF‐1, which stimulates EC proliferation and angiogenesis [55]. In ECs, IGF‐1 helps maintain their phenotype and function [138]. In an in vitro study, researchers found that IGF‐1 increases microvessel formation and significantly upregulates angiogenesis‐associated genes and proteins in ECs. Coculturing ECs and adipose‐derived stem cells (ASCs) amplified the proangiogenic effect of IGF‐1 [139]. Another in vitro study found that mouse ECs and ASCs increase the formation of blood vessels when cocultured as compared to mouse ECs cultured alone [140].

### 5.3. ECs in Breast Cancer and Obesity

A recent study identified a specialised subtype of breast capillary ECs known as lipid‐processing endothelial cells (LIPECs). These cells were characterised by their expression of key lipid metabolism markers, including fatty acid–binding Protein 4 and peroxisome proliferator–activated receptor gamma (PPAR‐*γ*), a transcription factor that regulates lipid uptake and storage. The LIPECs appear to contribute to lipid handling within the vascular microenvironment [141]. Notably, the study found that LIPECs were less abundant in breast cancer tissue compared to the adjacent nontumour region. Furthermore, LIPECs within tumour tissue exhibited elevated expression of Hes1, a transcriptional regulator known to suppress PPAR‐*γ* activity and influence vascular remodelling [141]. Since obesity is associated with increased circulating lipid levels and altered metabolic signalling [142], these findings raise the possibility that metabolic conditions may affect the presence or function of LIPECs. This could potentially disrupt lipid‐sensitive angiogenic pathways, thereby influencing the structure and function of blood vessels within the TME.

## 6. Pericytes

Pericytes are specialised cells located between the basement membrane and ECs of capillaries [143]. Together with the basement membrane and vascular smooth muscle cells, they contribute significantly to the structural integrity of human capillaries. Pericytes play a crucial role in forming the vascular wall, maintaining the vascular barrier and ensuring the stability of blood vessels [144–146]. The interaction between ECs and pericytes is essential to blood vessel formation and facilitates the exchange of chemical and mechanical signals between the two cell types [147]. Pericytes provide physical support and promote extracellular matrix synthesis [147]. The recruitment of pericytes is regulated by PDGF‐B [148].

During angiogenesis, pericytes separate from ECs in response to angiogenic signals, a process that also leads to basement membrane degradation [147]. Following EC proliferation and migration, pericytes are recruited to re‐establish vessel stability [147, 149]. The proper morphological formation of blood vessels depends on the interaction between ECs and pericytes. Thus, if it is disrupted, abnormalities in the vasculature can occur [147]. A study reported that ECs that express PDGF‐B facilitate the migration of PDGFR*β*‐positive pericytes to the ECs, promoting pericyte recruitment [150]. This finding further emphasises the critical role of angiogenic factors and EC–pericyte interactions in blood vessel formation.

### 6.1. Pericytes in Breast Cancer

Pericytes are involved in angiogenesis but are relatively understudied [151]. In tumours, pericytes move away from their usual positions, and the resulting abnormal vascular system is partly due to morphological and molecular changes in pericytes [152].

Breast cancer subtypes such as TNBC, Luminal A and Luminal B breast cancers exhibit distinct patterns of pericyte presence and behaviour around blood vessels, a concept known as pericyte heterogeneity [153]. A study analysing tissue microarrays from TNBC and Luminal A and B breast cancer samples focused on identifying pericytes using markers PDGFR*β* and desmin in proximity to CD31+ ECs. The results revealed that although TNBC tumours had a higher overall microvascular density, they showed significantly lower pericyte coverage, suggesting that many of these vessels were immature and poorly stabilised [154]. The composition of pericytes differed between the subtypes [154], with TNBC tumours predominantly containing PDGFR*β*+desmin− pericytes, which are considered less mature or stable [155]. Luminal tumours had higher proportions of PDGFR*β*+desmin+ and PDGFR*β*−desmin+ pericytes [154], which are both associated with vascular stability [155]. Given TNBC′s aggressive clinical behaviour [87], these differences in vascular architecture compared to the luminal tumours may indicate that its aggressiveness is partly driven by the quality of its blood vessels. The blood vessels created under tumorigenic angiogenic conditions tend to exhibit features of immature and highly permeable blood vessels, which are closely associated with the PDGF‐B/PDGFR*β* signalling pathway between ECs and pericytes [147]. This highlights the importance of pericytes and vascular structure in influencing breast cancer progression.

### 6.2. Pericytes in Obesity

In adipose tissue, pericytes play a crucial role in maintaining vascular integrity, facilitating angiogenesis and regulating blood flow [156]. In diet‐induced obese mice, elevated PDGF‐B secreted by macrophages activates the PDGFR*β* signalling pathway in pericytes. This activation leads to their detachment from blood vessels, which in turn promotes EC proliferation and angiogenesis [157–159]. Notably, an in vivo study using obese mice found that inhibition of PDGF‐B decreased pericyte detachment and led to decreased fat accumulation [160]. This indicates the essential role pericytes play in vascular remodelling and adipose tissue expansion. Table 1 summarises the factors and their effects reported in various studies.

**Table 1:** A table summarising the factors and effects observed in different studies.

Factor	Condition	Study/observation	Effect
VEGF	Breast cancer	↑ VEGF in tumours when compared to normal tissue [105, 109]	↑ Microvessel density, ↑ tumour growth, ↑ metastasis and poor prognosis [106–108]
	Obesity	↑ VEGF mRNA in vivo, ↑ vessel size and density [120, 121]	Hypervascularisation in adipose tissue [121]
	Breast cancer and obesity	↑ Microvessel density in breast tumours on HFD mice [64]	Obesity promotes angiogenesis via VEGF [121]

Leptin	Obesity	In vivo preadipocytes ↑ leptin secretion [34, 61]	Leptin ↑ VEGF transcription and angiogenesis [116]
Adiponectin	Obesity	↓ Adiponectin in obese mice [34, 61]	Loss of antiangiogenic balance in obesity [34, 61]
Hypoxia	Breast cancer	High levels of HIF‐1*α* and HIF‐2*α* [76–79]	Poor patient prognosis [87], ↑ tumour aggression [87] and ↑ VEGF‐A expression [88]
	Obesity	↓ pO_2_ in obese mice compared to lean mice [91]	Leads to adipose tissue hypoxia [91]
	Breast cancer and obesity	↑ HIF‐1 in adipocyte‐MCF‐7 coculture [98]	Hypoxia reduces hormone therapy efficacy and ↑ tumour progression [98]

FASN	Breast cancer and obesity	High‐calorie diets increase FASN [117, 118], which increases VEGF and VEGFR [119]	Lipid metabolism dysregulation may further drive angiogenesis in breast cancer [119]
ECs	Breast cancer	Irregular, leaky and poorly organised vessels [135]	Impaired vessel structure [135], leading to hypoxia, metastasis and treatment resistance [54]
	Obesity	Chronic inflammation, leading to EC activation and remodelling [3, 57]	Abnormal angiogenesis [3]

Pericytes	Breast cancer	Pericyte coverage ↓ in aggressive breast cancer subtypes [154]	Vascular stability differences in the different subtypes [154]
	Obesity	↑ PDGF‐B in vitro leading to ↑ angiogenesis [156–158]	Inhibition of PDGF‐B ↓ pericyte detachment [156–158] and fat accumulation [160]
	Breast cancer and obesity	PDGF‐B/PDGRF*β* pathway is found in breast cancer tissue and adipose tissue [147, 157–159]	Shared pericyte signalling abnormalities in breast cancer [147] and obesity [156–158]

## 7. Concluding Summary

This review is aimed at exploring the relationship between breast cancer and obesity from the perspective of angiogenesis. The evidence reviewed highlights the multifactorial interaction where obesity contributes to breast cancer progression by promoting angiogenesis, increasing tumour incidence and enhancing tumour aggressiveness. Angiogenesis plays a central role in tumour development, with the activation of the angiogenic switch enabling vascularisation that supports tumour progression and metastasis. However, the newly formed vessels are often dysfunctional, which perpetuates hypoxia and further malignancy. On the other hand, obesity promotes this process through hypertrophic tissue and altered adipokine profiles, which amplify proangiogenic signalling. These changes not only disrupt adipose tissue homeostasis but also create a TME conducive to cancer progression.

Hypoxia is a key driver of proangiogenic factor release and is commonly observed in both obesity and breast cancer due to their shared characteristic of rapid cellular proliferation. The overlap in hypoxic signalling pathways suggests a potential synergistic effect, where hypertrophic adipose tissue may exacerbate hypoxia within the TME. This heightened hypoxic state can further stimulate the release of proangiogenic factors, potentially worsening patient prognosis. Reviewed studies highlight that hypoxic signalling from adipose tissue can amplify tumour cell hypoxia and accelerate tumour progression. Intensified hypoxia may also contribute to treatment resistance and increased mortality. However, the findings were based on limited data, emphasising the need for more comprehensive in vivo studies across various timepoints, alongside clinical data sets.

The formation of new blood vessels is closely linked to elevated expression of VEGF. Reviewed studies reveal multiple mechanisms by which obesity enhances VEGF expression, including stimulation via FASN, leptin and hypoxia‐induced signalling pathways. These findings strongly suggest that increased adiposity contributes to heightened VEGF levels. Whilst VEGF is known to correlate with breast cancer progression and poor patient outcomes, the clinical data showing elevated serum VEGF levels were derived from breast cancer patients broadly, and not those specifically with obesity. Nonetheless, mechanistic overlap implies that obesity may further intensify VEGF‐driven angiogenesis, potentially promoting the growth of morphologically abnormal blood vessels, worsening hypoxia and perpetuating a self‐sustaining cycle of tumour growth and angiogenesis. To validate this link, future studies should analyse clinical data according to BMI and metabolic profiles and incorporate obese patient cohorts. Additionally, targeted in vivo studies using obese mouse models with breast cancer would provide deeper insight into the mechanistic link between adiposity, VEGF expression and tumour progression.

ECs and pericytes are crucial cells in angiogenesis regulation and vascular integrity. The abnormal vasculature is driven by abnormal EC and pericyte behaviours, which impair immune infiltration and therapeutic delivery. Obesity mirrors these vascular disruptions through chronic inflammation, altered metabolic signalling and pericyte detachment, further complicating the angiogenic balance. The identification of LIPECs introduced a novel link between lipid metabolism and vascular structure, suggesting that metabolic dysfunction may directly influence tumour angiogenesis. These shared mechanisms emphasise the importance of targeting ECs′ and pericytes′ metabolic pathways to improve vascular stability and therapeutic outcomes.

Despite these insights, gaps in the knowledge remain. Whilst preclinical models are strong, clinical studies are inconsistent, particularly regarding obesity‐mediated angiogenesis and its impact on breast cancer progression. Many studies lack longitudinal analysis of TME changes and angiogenic factors over time. Future research should focus on molecular biology and biomarker identification to enhance treatment strategies.

Understanding the complex interaction between obesity and breast cancer is crucial. It may pave the way for novel therapeutic approaches that combine antiangiogenic drugs with metabolic interventions or weight management strategies, which will ultimately improve patient outcomes.

## Author Contributions

Y.O.K.S., C.B., N.O. and M.A.A. contributed to the conception and design of the review. Y.O.K.S. performed the background data collection, analysis and interpretation. C.B., N.O. and M.A.A. provided supervision, reviewing and editing. M.A.A. was responsible for the critical review of the manuscript and the final approval. Y.O.K.S. wrote the initial draft of the manuscript.

## Funding

This study was supported by the South African Medical Research Council (10.13039/501100001322, A1H346) and the National Health Laboratory Service (10.13039/501100010753, GRANT004 94920).

## Disclosure

All authors have read and approved the final manuscript for publication.

## Ethics Statement

Ethical approval was granted by the ethics committee of the University of Pretoria with Ethics Number 449/2025.

## Conflicts of Interest

The authors declare no conflicts of interest.

## Data Availability

Data sharing is not applicable as no new data were generated, or the article describes entirely theoretical research.
